# Body Weight Reduction Results in Favorable Changes in Blood Pressure, Serum Lipids, and Blood Sugar in Middle-Aged Japanese Persons: A 5-Year Interval Observational Study of 26,824 Cases

**DOI:** 10.5539/gjhs.v7n5p159

**Published:** 2015-02-24

**Authors:** Nozomu Mandai, Kohei Akazawa, Nobuyuki Hara, Yoshio Ide, Koichi Ide, Ushio Dazai, Akiko Chishaki, Hiroaki Chishaki

**Affiliations:** 1 Department of Healthcare Management, College of Healthcare Management, 960-4 Takayanagi, Setaka-machi, Miyama-shi, Fukuoka, Japan; 2 Department of Medical Informatics and Statistics, Niigata University Graduate School of Medicine and Dental Sciences, 1-754 Asahimachi-dori, Chuo-ku, Niigata, Japan; 3 Fukuoka Foundation for Sound Health, 4-1-32 Tenjin, Chuo-ku, Fukuoka-shi, Fukuoka, Japan; 4 St. Mary’s Hospital, 422 Tsubukuhonmachi Kurume-shi, Fukuoka, Japan; 5 Faculty of Commerce Fukuoka University, 8-19-1 Nanakuma, Jonan-ku, Fukuoka, Japan; 6 Department of Health Sciences, Graduate School of Medical Sciences Kyushu University, 3-1-1 Maida-shi, Higashi-ku, Fukuoka, Japan

**Keywords:** metabolic syndrome, obesity, body mass index, body weight, Japan

## Abstract

**Objective:**

We investigated the relationships between body weight (BWt) and metabolic syndrome (MS) risk factors to elucidate the effect of BWt (ΔBWt) change and body mass index (BMI) on these factors in the Japanese population.

**Methods:**

Data were collected on MS-related parameters measured during two annual examinations of 16,640 men (mean age: 41.7±11.6 years) and 10,184 women (mean age: 45.0±12.2 years) without prior treatment of hypertension, diabetes mellitus, or dyslipidemia in 2006 and 2011 in Fukuoka, Japan. The subjects were divided into three groups according to BMI in 2006 (low, middle and high BMI) and into three groups according to change in BMI between 2006 and 2011 (decreased, stable, and increased BMI). Mean values for blood pressure (BP), systolic BP (SBP), diastolic BP (DBP), high-density lipoprotein cholesterol (HDL-C), low-density lipoprotein cholesterol (LDL-C), triglycerides (TG), hemoglobin A1c (HbA1c), and fasting blood glucose (FBG) for each group were determined by sex and subjected to statistical analysis for comparison.

**Results:**

High BMI (>26) was associated with higher SBP, LDL-C, FBG, and TG in both sexes. An increase ≥ 1.1 BMI units in 5 years was associated with increased DBP, LDL-C, TG, HbA1c, and FBG and decreased HDL-C. In contrast, decreased BMI was associated with decreased BP and LDL-C and increased HDL-C in both sexes, and decreased TG in men and FBG in women.

**Conclusions:**

Maintaining a desirable weight or losing weight may help prevent hypertension and MS, even in non-obese individuals.

## 1. Introduction

Obesity is associated with increased morbidity and mortality, including hypertension (HTN), diabetes (DM), dyslipidemia (DL), and renal disease (Nguyen, 2012; Yoon, 2006; Hsu, 2006; WHO, 2013). Both the prevalence of obesity and obesity-related diseases has been increasing worldwide (Siervo, 2014; Finucane, 2011). The World Health Organization (WHO) criteria for overweight and obesity are currently a body mass index (BMI) >25 and a BMI >30, respectively. However, these criteria have often been found unsuitable for Asian populations, as considerably different BMI distributions have been observed between Asian and non-Asian populations, among different Asian populations, and even within the same Asian population, including the Japanese population (WHO Expert Consultation, 2004; Wulan, 2010; Stevens, 2003; Kagawa, 2006; Examination Committee, 2002). According to the 2006 National Health and Nutrition Survey (NHNSJ, Table 1), population-wide average BMI values were lower in Japan than those of many countries in Western Europe or North America (Finucane, 2011; WHO technical report series, 2000), so WHO-defined obese patients are rarely observed at ordinary outpatient clinics in Japan. But Japan also faces an increase in overweight- and obesity-related health problems (Matsushita, 2004; Yoshiike, 2002; Liu, 1999). This may be due to the fact that weight gain in the Japanese results in more visceral fat accumulation, which leads to an earlier onset of metabolic syndrome (MS), DM, and HTN (Davis, 2013) than in other populations.

**Table 1:** BMI distribution in a representative sample of Japanese male and female participants of the 2006 Japan National Health and Nutrition Survey

BMI (kg/m^2^)	Total		Men		Women^a^	

	*N*	*%*	*N*	*%*	*N*	*%*
<15	6	0.1	4	0.1	2	0.1
15–15.9	21	0.3	11	0.4	10	0.3
16–16.9	91	1.3	25	0.8	66	1.8
17–17.9	230	3.4	68	2.2	162	4.3
18–18.9	428	6.3	149	4.8	279	7.4
19–19.9	611	8.9	185	6	426	11.4
20–20.9	741	10.8	286	9.2	455	12.1
21–21.9	857	12.5	325	10.5	532	14.2
22–22.9	767	11.2	389	12.6	378	10.1
23–23.9	773	11.3	407	13.2	366	9.8
24–24.9	656	9.6	362	11.7	294	7.9
25–25.9	518	7.6	295	9.5	223	6
26–26.9	381	5.6	220	7.1	161	4.3
27–27.9	252	3.7	127	4.1	125	3.3
28–28.9	166	2.4	80	2.6	86	2.3
29–29.9	113	1.7	56	1.8	57	1.5
30–30.9	83	1.2	42	1.4	41	1.1
31–31.9	52	0.8	22	0.7	30	0.8
32–32.9	38	0.6	17	0.5	21	0.6
33–33.9	19	0.3	9	0.3	10	0.3
34–34.9	16	0.2	7	0.2	9	0.2
≥35	19	0.3	7	0.2	12	0.3
Total	6838	100	3093	100	3745	100

*Note*. BMI: body mass index; N: number of subjects.

a Pregnant women were excluded.

Many studies conducted in western countries have indicated that body weight reduction improves response and outcome in treating HTN and other diseases (Winnicki, 2006; Fogari, 2010; Neter, 2003). However, as these studies only examined overweight or obese patients with a BMI >25 kg/m^2^, their findings might not be directly applicable to Asian populations with lower BMI profiles. As few studies have examined the effect of ΔBWt on patients with lower BMIs, little evidence has been accumulated. To fill this research gap, this study retrospectively examined a large group of Japanese persons to elucidate the relationship between BWt and MS risk factors and analyzed the effect of ΔBWt on these factors.

## 2. Methods

### 2.1 Study Design

A retrospective, observational, comparative design was used to perform this non-intervention study.

### 2.2 Study Population

The Fukuoka Foundation for Sound Health, Fukuoka, Japan, conducts various medical examinations of over 200,000 people annually. Medical records contained in the Fukuoka Foundation database were reviewed to select participants who met the inclusion criteria of having undergone medical examinations in both 2006 and 2011 during which height, BWt, and blood pressure (BP) were measured and did not meet the exclusion criteria of having undergone treatment for HTN, DL, or DM until 2011. Records of 16,640 men and 10,184 women were extracted anonymously and stored in an isolated computer for off-line analysis. Age, sex, weight, height, BMI, systolic BP (SBP), diastolic BP (DBP), low-density lipoprotein cholesterol (LDL-C) level, high-density lipoprotein cholesterol (HDL-C) level, triglyceride (TG) level, fasting blood glucose (FBG) level, and hemoglobin A1c (HbA1c) were examined. Because the direct measurement of serum LDL-C was not available in 2006, LDL-C values were calculated using Friedwald’s equation as follows: Total cholesterol – HDL-C – TG / 5.33 (Miller, 2010). Because of the efficacy of this equation, patients with a calculated LDL-C <50 and >350 mg/dl were excluded (Miller, 2010).

### 2.3 Ethical Considerations

All participants provided informed consent for the anonymous analysis of their personal data at interviews prior to the medical examinations. The Internal Review Board of Medical Ethics at the College of Healthcare Management approved this study.

### 2.4 Evaluation of Parameters and Methods

#### 2.4.1 Subject Categorization

The study began with categorization of the subjects into BMI groups according to the 2006 data. Instead of categorizing the participants as underweight (BMI <18.50), normal weight (BMI 18.50–24.99), or overweight or obese (BMI ≥25) by WHO BMI criteria, we divided subjects into three groups (low BMI [BMI <21.1; 4066 men and 4971 women], medium BMI [BMI 21.1–23.8; 5998 men and 3116 women], and high BMI [BMI >23.8; 6576 men and 2097 women]), in order to show the relative relationships with BWt and its change within the participants. Although our categorization was arbitrary, medium BMI group of each sex contained overall means of BMI. The means of the groups were then statistically compared by sex.

#### 2.4.2 Evaluation of Effect of Change in Body Weight Between 2006 and 2011

**Figure 1:**
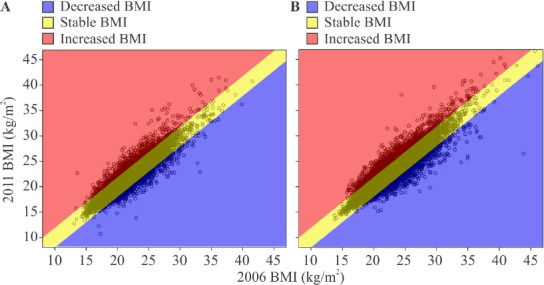
Change in body mass index (BMI) between 2006 and 2011 by sex *Note*. Each circle represents one subject. Subjects were divided into the decreased BMI (ΔBMI ≤ -1.1; blue), stable BMI (ΔBMI -1.1–1.1; yellow), and increased BMI (ΔBMI ≥1.1) groups based on change in BMI; (A) Male subjects; (b) Female subjects.

To examine the effect of change in BWt (ΔBMI) between 2006 and 2011 on the parameters examined, the subjects were categorized into the decreased BMI (ΔBMI ≤-1.1; 2319 men and 1483 women), stable BMI (ΔBMI -1.1–1.1; 5998 men and 3116 women), and increased BMI (ΔBMI ≥1.1; 6576 men and 2097 women) groups. Fig. 1 shows the distribution of BMIs in 2011 plotted against BMIs in 2006 and gives the visual image of the changes of BMIs in 5 years. Men and women were grouped according to the same criterion because it seemed to satisfy our common sense of gaining/losing weight among Japanese. The mean values of the parameters of the groups were then statistically compared.

### 2.5 Statistical Analysis

#### 2.5.1 Software and Level of Significance

We used Statistical Package for the Social Sciences (SPSS) ver.17.0 (SPSS, Inc., Chicago, IL, USA) for all statistical analysis. Statistical significance level was set at p<0.05.

#### 2.5.2 Cross-Sectional Comparison

Cross-sectional comparison began with performance of the Levene test to determine the equality of variance among the BMI groups. On the basis of the results, the means of the parameters of the BMI groups were compared using either one-way ANOVA or Welch’s test. To correct for the level of significance, the Games–Howell test was performed to compare the mean values among the groups of each sex. Changes in the means of the parameters in each group were tested with the paired t-test.

## 3. Results

### 3.1 Distribution of Parameters

**Figure 2:**
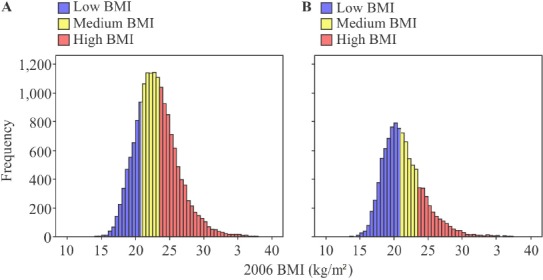
Number of subjects in each body mass index (BMI) group by sex *Note*. Low BMI: BMI <20.9 (blue); medium BMI: BMI 20.9–23.5 (yellow): high BMI: BMI >23.5 (red); A: Male subjects; B: Female subjects.

Table 2 shows the distribution of the parameters of the 16,640 men and 10,184 women examined in this study. Compared to the female subjects, the male subjects were slightly younger (mean age 45.0±12.2 years vs. 41.7±11.6 years; p<0.001) and had a higher mean BMI (21.4±3.1 vs. 23.1±3.2; p<0.001). For both sexes, the distribution of BMI was similar to that found in a 2006 NHNSJ survey, with few subjects having a BMI >30 (Figure 2).

**Table 2:** Subject characteristics and serum parameters at baseline

Parameter (unit)	Men			Women		

	*N*	*Mean*	*SD*	*N*	*Mean*	*SD*
Age (years)	16640	41.7	11.6	10184	45.0	12.2
Height (cm)	16640	169.8	6.0	10184	156.2	5.7
Weight (kg)	16640	66.5	13.3	10184	52.2	8.0
BMI (kg/m^2^)	16640	23.1	3.2	10184	21.4	3.1
SBP (mm Hg)	16640	123.0	14.5	10184	115.2	15.0
DBP (mm Hg)	16640	75.9	10.3	10184	70.3	9.7
HDL-C (mmol/l)	16058	1.4	0.3	9497	1.7	0.3
LDL-C (mmol/l)	15809	3.2	0.8	9397	3.1	0.8
TG (mmol/l)	11000	1.4	1.2	7338	0.8	0.5
FBG (mmol/l)	10465	5.2	0.5	7383	5.0	0.5
HbA1c (%)	4732	5.50	0.69	3519	5.51	0.47

*Note*. N: number of subjects after selection criteria were applied; SD: standard deviation; BMI: body mass index; SBP: systolic blood pressure; DBP: diastolic blood pressure; HDL-C: high-density lipoprotein cholesterol; LDL-C: low-density lipoprotein cholesterol; TG: triglycerides; HbA1c: hemoglobin A1c; FBG: fasting blood glucose.

### 3.2 Cross Sectional Analysis of 2006 Data

Table 3 shows the mean and standard deviation (SD) values and Table 4 shows the results of the analysis of changes in the parameters of the BMI groups of each sex. Mean values of every parameter differed among the three BMI groups of each sex. For the male subjects, further comparison revealed that the mean values of every parameter except for the mean HbA1c level significantly differed between the low and medium BMI groups. In all groups, the mean SBP and mean DBP values were within normal range. However, the low BMI groups of both sexes had the lowest values and the high BMI groups had the highest values. The differences among the groups were statistically significant. The same trends were seen regarding the LDL-C, TG, and FBG levels in both sexes and the HbA1c in women. As to HbA1c in the male subjects, the high BMI group had a higher mean than the other two groups. And for the mean HDL-C levels, the low BMI groups of both sexes had the highest values, while the high BMI groups of both sexes had the lowest values. As it is generally understood that higher BP, LDL-C, TG, HbA1c, and FBG levels and lower HDL-C levels are unfavorable, the results indicate that higher BMI was related to unfavorable values of these parameters.

**Table 3:** Comparison of mean serum parameter values in BMI groups at baseline by sex

Parameter (unit)	BMI group										Group difference		

	*Low BMI*			*Medium BMI*			*High BMI*			*ANOVA p-value*	*Specific group difference^a^*		
									
	N	Mean	SD	N	Mean	SD	N	Mean	SD		L vs. M	M vs. H	H vs. L
Men														
SBP (mm Hg)	4066	118.5	13.9	5998	121.9	13.5	6576	126.8	14.7	<0.0001	M	H	H
DBP (mm Hg)	4066	72.5	9.6	5998	74.8	9.7	6576	79.0	10.3	<0.0001	M	H	H
HDL-C (mmol/l)	4016	1.6	0.4	5794	1.5	0.3	6248	1.3	0.3	<0.0001	L	M	L
LDL-C (mmol/l)	3941	2.8	0.7	5715	3.1	0.8	6153	3.4	0.9	<0.0001	M	H	H
TG (mmol/l)	2512	1.0	0.7	4061	1.3	1.0	4427	1.7	1.4	<0.0001	M	H	H
FBG (mmol/l)	2333	5.1	0.6	3836	5.2	0.5	4296	5.3	0.7	<0.0001	M	H	H
HbA1c (%)	1051	5.39	0.59	1695	5.42	0.59	1986	5.63	0.80	<0.0001	n.s.	H	H
Women														
SBP (mm Hg)	4971	111.3	13.8	3116	116.4	14.4	2097	122.7	15.6	<0.0001	M	H	H
DBP (mm Hg)	4971	68.0	9.0	3116	70.8	9.3	2097	74.9	10.2	<0.0001	M	H	H
HDL-C (mmol/l)	4761	1.8	0.3	2870	1.7	0.3	1866	1.6	0.3	<0.0001	L	M	L
LDL-C (mmol/l)	4706	2.9	0.7	2842	3.2	0.8	1849	3.4	0.8	<0.0001	M	H	H
TG (mmol/l)	3652	0.7	0.4	2257	0.9	0.6	1429	1.1	0.7	<0.0001	M	H	H
FBG (mmol/l)	3538	4.9	0.4	2340	5.0	0.4	1505	5.2	0.6	<0.0001	M	H	H
HbA1c (%)	1549	5.41	0.35	1132	5.53	0.47	838	5.66	0.60	<0.0001	M	H	H

*Note*. N: number of subjects after selection criteria were applied; SD: standard deviation; BMI: body mass index (kg/m^2^); SBP: systolic blood pressure; DBP: diastolic blood pressure; HDL-C: high-density lipoprotein cholesterol; LDL-C: low-density lipoprotein cholesterol; TG: triglycerides; HbA1c: hemoglobin A1c; FBG: fasting blood glucose; low BMI: BMI <20.9; medium BMI: BMI 20.9–23.5; high BMI: BMI ≥23.5; ANOVA: analysis of variance; L vs. M: comparison of low BMI and medium BMI groups; M vs. H: comparison of medium and high BMI groups; H vs. L: comparison of high and low BMI groups;

a Games–Howell comparison was used to analyze the differences between the means of the BMI groups; L: low BMI group is significantly greater at p<0.0001; M: medium BMI group is significantly greater at p<0.0001; H: high BMI group is significantly greater at p<0.0001; n.s.: not significant.

**Table 4:** Mean serum parameter values in BMI groups by sex in 2006 and 2011

Parameter (unit)	BMI group																	

	Decreased BMI					*p*-value^a^	Stable BMI					*p*-value^a^	Increased BMI					*p*-value^a^
		
		2006		2011				2006		2011				2006		2011		
	N	Mean	SD	Mean	SD		N	Mean	SD	Mean	SD		N	Mean	SD	Mean	SD	
Men																		

SBP (mm Hg)	2319	127•0	15•6	120•5	15•0	<0•0001^b^	10461	122•7	14•2	120•4	14•8	<0•0001	3860	121•3	13•4	121•9	13•9	0•005^c^
DBP (mm Hg)	2319	78•9	10•8	74•9	11•5	<0•0001	10461	75•8	10•1	74•7	11•1	<0•0001	3860	74•4	10•1	75•8	11•3	<0•0001
HDL-C (mmol/l)	2221	1•4	0•3	1•6	0•4	<0•0001	10087	1•5	0•4	1•6	0•4	<0•0001	3750	1•4	0•3	1•4	0•3	<0•0001
LDL-C (mmol/l)	2188	3•3	0•9	3•0	0•8	<0•0001	9928	3•1	0•8	3•1	0•8	<0•0001	3693	3•1	0•8	3•3	0•8	<0•0001
TG (mmol/l)	1597	1•6	1•1	1•2	0•8	<0•0001	7039	1•4	1•1	1•4	1•0	n.s.	2364	1•3	1•3	1•6	1•3	<0•0001
FBG (mmol/l)	1489	5•4	1•0	5•4	1•5	n.s.	6733	5•2	0•6	5•2	0•8	<0•0001	2243	5•1	0•5	5•2	0•6	<0•0001
HbA1c (%)	764	5•72	0•88	5•75	1•01	n.s.	3085	5•47	0•63	5•58	0•65	<0•0001	883	5•43	0•69	5•61	0•64	<0•0001

Women																		

SBP (mm Hg)	1483	119•1	16•3	113•8	16•6	<0•0001	6604	114•9	14•8	112•7	15•4	<0•0001	2097	113•6	14•4	114•3	14•8	0•023
DBP (mm Hg)	1483	72•1	10•5	68•6	10•9	<0•0001	6604	70•1	9•5	68•1	10•9	<0•0001	2097	69•6	9•6	69•6	11•2	n.s.
HDL-C (mmol/l)	1342	1•7	0•4	1•9	0•4	<0•0001	6164	1•7	0•3	1•8	0•4	<0•0001	1991	1•7	0•3	1•8	0•4	<0•0001
LDL-C (mmol/l)	1330	3•3	0•8	3•0	0•8	<0•0001	6106	3•1	0•8	3•0	0•8	<0•0001	1961	3•0	0•8	3•1	0•8	<0•0001
TG (mmol/l)	1024	1•0	0•6	0•8	0•5	<0•0001	4799	0•8	0•5	0•9	0•5	<0•0001	1515	0•8	0•5	1•0	0•6	<0•0001
FBG (mmol/l)	1052	5•1	0•7	5•0	1•1	<0•0001	4854	5•0	0•5	5•0	0•5	<0•0001	1477	5•0	0•4	5•0	0•5	<0•0001
HbA1c (%)	592	5•64	0•67	5•65	0•65	n.s.	2341	5•50	0•40	5•58	0•44	<0•0001	586	5•43	0•45	5•55	0•42	<0•0001

*Note*. N: number of subjects after selection criteria were applied; SD: standard deviation; BMI: body mass index (kg/m^2^); SBP: systolic blood pressure; DBP: diastolic blood pressure; HDL-C: high-density lipoprotein cholesterol; LDL-C: low-density lipoprotein cholesterol; TG: triglycerides; HbA1c: hemoglobin A1c; FBG: fasting blood glucose; decreased BMI: ΔΔBMI ≥-1.1; stable BMI: ΔBMI -1.1–1.1; increased BMI: ΔBMI ≥ 1.1; ΔBMI = 2011 BMI – 2006 BMI;

a Paired t-test was used to analyze significant differences between mean serum parameter values in 2006 and 2011;

b blue highlighting indicates a significant decrease in the mean serum parameter value;

c orange highlighting indicates a significant increase in the mean serum parameter value; n.s., not significant

### 3.3 Evaluation of Changes of Parameters and BMI

Table 4 shows changes of parameters and BMI values using the paired t-test. In men, means of SBP and means of DBP of decreased and stable BMI groups significantly decreased from 2006 to 2011. In contrast, mean of SBP and mean of DBP of the increased BMI group significantly increased over this period. In women, means of SBP and means of DBP of the decreased and stable BMI groups significantly decreased, while mean of SBP of the increased BMI group increased. But mean of DBP of increased BMI group did not change significantly.

### 3.4 Changes in HDL-C and LDL-C Levels

The mean HDL-C levels of all BMI groups of both sexes significantly increased. The largest increases in the mean HDL-C levels of both sexes occurred in the decreased BMI groups, suggesting that BWt reduction has a favorable effect on serum HDL-C levels in both sexes. In contrast, the mean LDL-C levels significantly deceased in the decreased and stable BMI groups in both sexes, but significantly rose in the increased BMI groups in both sexes. Unfavorable changes in serum LDL-C levels were thus only observed in the increased BMI groups of both sexes.

### 3.5 Changes in TG Levels

In men, the mean TG level decreased significantly in the decreased BMI group, did not change significantly in the stable BMI group, and increased significantly in the increased BMI group. In women, the mean TG level decreased significantly in the decreased BMI group, but increased significantly in the stable and increased BMI groups. These results indicate that BMI decrease both in men and in women has a favorable effect on serum TG levels.

### 3.6 Changes in FBG Levels and HbA1c

In men, the mean FBG level of the decreased BMI group did not change significantly, whereas the mean FBG levels of the stable and increased BMI groups increased significantly. Likewise, while the mean HbA1c of the decreased BMI group of men did not change significantly, the mean HbA1c of the stable BMI and increased BMI groups increased significantly. In women, the mean FBG levels of the decreased BMI and stable BMI groups significantly deceased, while the mean FBG level of the increased BMI group increased significantly. Although the mean HbA1c of the decreased BMI group of women did not change significantly, the mean HbA1c of the stable and increased BMI groups increased significantly.

## 4. Discussion

To retrospectively examine the relationship between BWt and MS-related parameters in apparently healthy Japanese and determine the effect of BWt changes on these parameters over a 5-year observation period, we conducted a non-interventional study using data from the health records of nearly 27,000 individuals between 2006 and 2011. Our present findings may be applied to other Asian populations for which WHO criteria for obesity have been reported to be unfit (WHO Expert Consultation, 2004; Wulan, 2010; Stevens, 2003; Kagawa, 2006; Examination Committee, 2002).

### 4.1 Results of Comparison of Parameters by BMI

Simple comparison among the three BMI groups of each sex revealed that high BMI was related to unfavorable mean values of every parameter, regardless of the fact that the mean values remained within normal ranges. Although we did not conduct linear regression analysis that might have provided more descriptive, quantitative explanations of the relationships between BMI and the parameters, our results supported the simple message that being overweight is detrimental to serum lipid profile and blood sugar by changing blood pressure. One of our aims was to provide participants with a simple message for their daily lives, which we believe was successfully achieved. Of note, the division of subjects into low, medium, and high BMI groups according to the actual distribution of BMI for analysis was simple and quite useful.

### 4.2 Effect of Change in BMI on MS-Related Parameters

We set relatively arbitrary criteria for grouping subjects according to BMI changes: decreased BMI (ΔBMI ≤ −1.1), stable BMI (−1.1 < ΔBMI < 1.1), and increased BMI (1.1 ≤ ΔBMI), where ΔBMI was the difference in BMI between 2006 and 2011. Stayed BMI meant that BWt stayed within ±3.18 kg of the BWt in 2006 for a person who is 170 cm tall. Categorization of “stable BMI” fell under the category of “stable BMI” that had been defined as no more than ± 1.2 kg/m^2^ change in BMI over 6 years in previous reports (Lloyd-Jones, 2007; Korkeila, 2009). Use of this criterion appeared reasonable when assessing BWt changes over a 5-year period in the current study.

### 4.3 Changes in BPs Levels

Findings of the intergroup analysis of BP values were quite interesting. Mean BP in both the decreased BMI and stable BMI groups were markedly reduced by more than 2 mmHg—this is the magnitude of BP change that has been reported to dramatically reduce the risk of comorbidities due to hypertension. These findings suggest that losing or at least not gaining weight may keep BP from rising in both sexes, whereas gaining more than 1.1 BMI unit may raise SBP in both sexes and DBP in men.

These findings appear to contradict the current understanding of the relationship between age and BP to some extent––particularly, the belief that BP tends to increase with age. The results of the Framingham Heart Study, which indicated that individuals tended to experience a linear increase in SBP between the ages of 30 and 84 years (Franklin, 1997), were supported by the finding that prevalence of hypertension increases with age in both the United States and Japan (Yoon, 2012) (Table 5).

**Table 5:** Age distribution of hypertensive and non-hypertensive individuals in a representative sample of participants in the 2006 Japan National Health and Nutrition Survey

Age (years)	Total	Hypertensive		Non-hypertensive	
	
	N	N	%	N	%
20–29	297	10	3.4	287	96.7
30–39	614	60	9.8	554	90.2
40–49	593	130	21.9	463	78.1
50–59	907	428	47.2	479	52.8
60–69	972	597	61.4	375	38.5
≥70	1135	821	72.3	314	27.6
Total	4518	2046	45.3	2472	54.7

One explanation for this discrepancy between the present and previous findings is that 5 years is not a sufficient period for adequately clarifying the relationship between age and BP changes, which may require 15 to 20 years of observation. Another explanation is that BWt reduction contributed to SBP decrease or prevention of SBP increase, and that stable BMI affected changes in BP to some extent. While 5 years is admittedly a relatively short duration for examination, our findings nevertheless suggest that maintaining a stable weight assists in decreasing BP over 5 years, and possibly 10 years. This implication is clinically significant, as delaying the onset of hypertension for as long as 10 years would greatly benefit both individuals and communities. Previous studies have reported a significant relationship between BP and BMI (Dyer, 1989), and we believe our findings support these results.

### 4.4 Changes in HDL-C Levels

In all BMI groups of both sexes, the mean serum HDL-C levels increased significantly from 2006 to 2011, with the largest increases occurring in the decreased BMI groups. Results of several previous studies of the relationship between HDL-C levels and age were not uniformly conclusive, with some finding that HDL levels declined with age and others noting no marked changes (Ferrara, 1997; Criqui, 1983; Ettinger, 1992). While the reason for the increase in HDL-C levels of all groups is unclear, it is noteworthy that the largest increases occurred in the decreased BMI groups of both sexes. This finding, in addition to the finding that the low BMI groups of both sexes had the highest mean HDL-C levels, indicates that maintaining low BMI and reducing BMI had favorable effects on HDL-C levels of the population examined.

Previous studies have suggested that weight control may help prevent arteriosclerotic disease (Yatsuya, 2010) and that HDL-C markedly influences the risk of arteriosclerotic disease (Tanabe, 2010). We believe our findings support these results.

### 4.5 Changes in LDL-C Levels

In both sexes, increased BWt was associated with increased serum LDL-C levels and decreased BWt with decreased levels. The 2006 NHNSJ study reported that total serum cholesterol levels and percentages of participants who were aware of having high serum cholesterol levels increased in both sexes over the 10-year study period. Our findings suggest that informing individuals about the benefit of BWt reduction in preventing a rise in total serum cholesterol levels and in reducing LDL-C levels may encourage them to stay in shape and lose weight. While we did not inquire about participants’ daily activities, dietary habits, or efforts towards losing weight or reducing LDL-C, BWt reduction was associated with a decrease in LDL-C, and this finding may encourage ordinary people to stay in shape and lose weight. Previous studies have suggested that weight control may help prevent arteriosclerotic disease (Yatsuya, 2010) and that LDL-C markedly influences the risk of arteriosclerotic disease (Karthikeyan, 2009; Sever, 2003; Okazaki, 2004); we believe our findings support these results.

### 4.6 Changes in FBG and HbA1c

Although not as dramatic as their effect on SBP and LDL-C levels, increases in BWt clearly had minor but still unfavorable effects on both FBG and HbA1c levels. While the results did not clarify the means of change or amount of change necessary to do damage, they do suggest that negligible changes in BWt can lead to non-negligible changes in FBG and HbA1c levels in the long term. From the viewpoint of early intervention for prevention, increases in these parameters should be regarded as warning signs of MS and MS-related disorders. Previous studies have reported a significant relationship between the risk of diabetes and BMI (Boffetta, 2011); we believe our findings support these results.

### 4.7 Study Limitations

Several limitations in our study warrant mention. First, we used relatively arbitrary criteria to divide subjects into groups for analysis. However, given that the study results largely agreed with previous findings, we feel these criteria were appropriate. Further, as the WHO criteria for obesity have been reported to be unsuitable for the Asian population (WHO Expert Consultation, 2004; Stevens, 2003; Kagawa, 2006; Examination Committee, 2002), we feel that our usage of unreported but appropriate criteria was well founded and that our criteria might be applicable to other Asian populations with BMI distributions similar to that of the Japanese. Second, because we only examined BWt, the effects of other factors on parameter levels such as daily exercise were not taken into account. Third, we conducted relatively little follow-up over the course of the study—we only examined the parameters at baseline and at the end of the study. More frequent follow-up should be conducted in future studies to provide an accurate picture of any changes. Fourth, regarding descriptive data, we only examined age and sex; future studies should incorporate more variables to further enrich the data set.

Regardless of these limitations, we believe that our study involving a relatively large number of subjects and parameters compared with previous studies of the Japanese population with similar results (Ishikawa-Takata, 2002; Hirose, 2000; Nagaya, 2005) has valid and reliable findings.

## 5. Conclusions

Our findings suggest that reducing BWt can help to control BP, serum lipid profiles, and blood sugar levels, while gaining BWt has unfavorable effects on these parameters. In the present study, the values of the examined parameters remained within the normal range in subjects despite BWt changes, although the long-term effects of these changes remain to be elucidated through long-term follow-up studies. Taken together, these findings suggest that maintaining a BWt that is appropriate for an individual’s height or decreasing BWt has favorable effects on MS risk factors. These findings can be applied to clinical practice for the prevention and management of MS and MS-related disorders to benefit the Japanese population and potentially other populations as well.

## References

[ref1] Boffetta P, McLerran D, Chen Y, Inoue M, Sinha R, He J, Potter J.D (2011). Epub 2011 Jun 22. Body mass index and diabetes in Asia: a cross-sectional pooled analysis of 900,000 individuals in the Asia cohort consortium. PLoS One.

[ref2] Criqui M. H, Frankville D. D, Barrett-Connor E, Klauber M. R, Holdbrook M. J, Turner J. D (1983). Change and correlates of change in high and low density lipoprotein cholesterol after six years: a prospective study. American Journal of Epidemiology.

[ref3] Davis J, Juarez D, Hodges K (2013). Relationship of ethnicity and body mass index with the development of hypertension and hyperlipidemia. Ethnicity & Disease.

[ref4] Dyer A. R, Elliott P (1989). The INTERSALT study: relations of body mass index to blood pressure. INTERSALT Co-operative Research Group. Journal of Human Hypertension.

[ref5] Ettinger W. H, Wahl P. W, Kuller L. H, Bush T. L, Tracy R. P, Manolio T. A, O’Leary D. H (1992). Lipoprotein lipids in older people. Results from the Cardiovascular Health Study. The CHS Collaborative Research Group. Circulation.

[ref6] Examination Committee of Criteria for ‘Obesity Disease’ in Japan, Japan Society for the Study of Obesity (2002). New criteria for ‘obesity disease’ in Japan. Circulation Journal.

[ref7] Ferrara A, Barrett-Connor E, Shan J (1997). Total, LDL, and HDL cholesterol decrease with age in older men and women. The Rancho Bernardo Study 1984-1994. Circulation.

[ref8] Finucane M. M, Stevens G. A, Cowan M. J, Danaei G, Lin J. K, Paciorek C. J, Ezzati M (2011). National, regional, and global trends in body-mass index since 1980: systematic analysis of health examination surveys and epidemiological studies with 960 country-years and 9.1 million participants. Lancet.

[ref9] Fogari R, Zoppi A, Corradi L, Preti P, Mugellini A, Lazzari P, Derosa G (2010). Effect of body weight loss and normalization on blood pressure in overweight non-obese patients with stage 1 hypertension. Hypertension Research.

[ref10] Franklin S. S, Gustin W. T, Wong N. D, Larson M. G, Weber M. A, Kannel W. B, Levy D (1997). Hemodynamic patterns of age-related changes in blood pressure. The Framingham Heart Study. Circulation.

[ref11] Hirose H, Saito I, Tsujioka M, Kawabe H, Saruta T (2000). Effects of body weight control on changes in blood pressure: three-year follow-up study in young Japanese individuals. Hypertension Research.

[ref12] Hsu C. Y, McCulloch C. E, Iribarren C, Darbinian J, Go A. S (2006). Body mass index and risk for end-stage renal disease. Annals of Internal Medicine.

[ref13] Ishikawa-Takata K, Ohta T, Moritaki K, Gotou T, Inoue S (2002). Obesity, weight change and risks for hypertension, diabetes and hypercholesterolemia in Japanese men. European Journal of Clinical Nutrition.

[ref14] Kagawa M, Kerr D, Uchida H, Binns C. W (2006). Differences in the relationship between BMI and percentage body fat between Japanese and Australian-Caucasian young men. British Journal of Nutrition.

[ref15] Karthikeyan G, Teo K. K, Islam S, McQueen M. J, Pais P, Wang X, Yusuf S (2009). Lipid profile, plasma apolipoproteins, and risk of a first myocardial infarction among Asians: an analysis from the INTERHEART study. Journal of the American College of Cardiology.

[ref16] Korkeila M, Rissanen A, Sorensen T. I, Kaprio J (2009). BMI, weight stability and mortality among adults without clinical co-morbidities: a 22-year mortality follow-up in the Finnish twin cohort. Obesity Facts.

[ref17] Liu L, Choudhury S. R, Okayama A, Hayakawa T, Kita Y, Ueshima H (1999). Changes in body mass index and its relationships to other cardiovascular risk factors among Japanese population: results from the 1980 and 1990 national cardiovascular surveys in Japan. Journal of Epidemiology.

[ref18] Lloyd-Jones D. M, Liu K, Colangelo L. A, Yan L. L, Klein L, Loria C. M, Savage P (2007). Consistently stable or decreased body mass index in young adulthood and longitudinal changes in metabolic syndrome components: the Coronary Artery Risk Development in Young Adults Study. Circulation.

[ref19] Matsushita Y, Yoshiike N, Kaneda F, Yoshita K, Takimoto H (2004). Trends in childhood obesity in Japan over the last 25 years from the national nutrition survey. Obesity Reviews.

[ref20] Miller W. G, Myers G. L, Sakurabayashi I, Bachmann L.M, Caudill S.P, Dziekonski A, Remaley A. T (2010). Seven direct methods for measuring HDL and LDL cholesterol compared with ultracentrifugation reference measurement procedures. Clinical Chemistry.

[ref21] Nagaya T, Yoshida H, Takahashi H, Kawai M (2005). Increases in body mass index, even within non-obese levels, raise the risk for Type 2 diabetes mellitus: a follow-up study in a Japanese population. Diabetic Medicine.

[ref22] Neter J. E, Stam B. E, Kok F. J, Grobbee D. E, Geleijnse J. M (2003). Influence of weight reduction on blood pressure: a meta-analysis of randomized controlled trials. Hypertension.

[ref23] Nguyen T, Lau D. C (2012). The obesity epidemic and its impact on hypertension. Canadian Journal of Cardiology.

[ref24] Okazaki S, Yokoyama T, Miyauchi K, Shimada K, Kurata T, Sato H, Daida H (2004). Early statin treatment in patients with acute coronary syndrome: demonstration of the beneficial effect on atherosclerotic lesions by serial volumetric intravascular ultrasound analysis during half a year after coronary event; the ESTABLISH study. Circulation.

[ref25] Sever P.S, Dahlöf B, Poulter N.R, Wedel H, Beevers G, Caulfield M, Ostergren J (2003). Prevention of coronary and stroke events with atorvastatin in hypertensive patients who have average or lower-than-average cholesterol concentrations, in the Anglo-Scandinavian cardiac outcomes trial-lipid lowering arm (ASCOT-LLA): a multicentre randomised controlled trial. Lancet.

[ref26] Siervo M, Montagnese C, Mathers J. C, Soroka K. R, Stephan B. C, Wells J. C (2014). Sugar consumption and global prevalence of obesity and hypertension: an ecological analysis. Public Health Nutrition.

[ref27] Stevens J, Nowicki E. M (2003). Body mass index and mortality in asian populations: implications for obesity cut-points. Nutrition Reviews.

[ref28] Tanabe N, Iso H, Okada K, Nakamura Y, Harada A, Ohashi Y, Ueshima H (2010). Circulation Journal, 74(7):1346-56. Epub 2010 Jun 4. Serum total and non-high-density lipoprotein cholesterol and the risk prediction of cardiovascular events - the JALS-ECC -. Japan Arteriosclerosis Longitudinal Study Group.

[ref29] WHO Obesity and overweight. Fact sheet, No. 311.

[ref30] WHO Expert Consultation (2004). Appropriate body-mass index for Asian populations and its implications for policy and intervention strategies. Lancet.

[ref31] WHO technical report series (2000). Obesity: preventing and managing the global epidemic. Report of a WHO consultation.

[ref32] Winnicki M, Bonso E, Dorigatti F, Longo D, Zaetta V, Mattarei M, Palatini P (2006). Effect of body weight loss on blood pressure after 6 years of follow-up in stage 1 hypertension. American Journal of Hypertension.

[ref33] Wulan S. N, Westerterp K. R, Plasqui G (2010). Ethnic differences in body composition and the associated metabolic profile: a comparative study between Asians and Caucasians. Maturitas.

[ref34] Yatsuya H, Toyoshima H, Yamagishi K, Tamakoshi K, Taguri M, Harada A, Ueshima H (2010). Circulation: Cardiovascular Quality and Outcomes, 3(5):498-505. Epub 2010 Aug 10. Body mass index and risk of stroke and myocardial infarction in a relatively lean population: meta-analysis of 16 Japanese cohorts using individual data. Japan Arteriosclerosis Longitudinal Study (JALS) group.

[ref35] Yoon K. H, Lee J. H, Kim J. W, Cho J. H, Choi Y. H, Ko S. H, Son H.Y (2006). Epidemic obesity and type 2 diabetes in Asia. Lancet.

[ref36] Yoon S. S, Burt V, Louis T, Carroll M. D (2012). Hypertension among adults in the United States, 2009-2010. NCHS Data Brief.

[ref37] Yoshiike N, Seino F, Tajima S, Arai Y, Kawano M, Furuhata T, Inoue S (2002). Twenty-year changes in the prevalence of overweight in Japanese adults: the National Nutrition Survey 1976-95. Obesity Reviews.

